# Correction: Interactions between marmatite and bornite during the oxidative dissolution process in abiotic and biotic systems

**DOI:** 10.1039/c9ra90072a

**Published:** 2019-10-17

**Authors:** Yanjun Zhang, Hongbo Zhao, Yisheng Zhang, Lu Qian, Luyuan Zhang, Xiaoyu Meng, Xin Lv, Pillai Abhilash, Hussnain Ahmed Janjua, Guanzhou Qiu

**Affiliations:** School of Minerals Processing & Bioengineering, Central South University No. 932 Lushan South Road, Yuelu District Changsha Hunan 410083 China alexandercsu@126.com; Key Lab of Biohydrometallurgy of Ministry of Education Changsha Hunan China; CSIR – National Metallurgical Laboratory Jamshedpur 831007 India; Atta-ur-Rahman School of Applied Biosciences, National University of Sciences and Technology (NUST) Sector H-12 Islamabad Pakistan

## Abstract

Correction for ‘Interactions between marmatite and bornite during the oxidative dissolution process in abiotic and biotic systems’ by Yanjun Zhang *et al.*, *RSC Adv.*, 2019, **9**, 26609–26618.

Hussnain Ahmed Janjua's name was incorrectly spelled in the published article; the corrected author list is shown here.

The Royal Society of Chemistry apologises for these errors and any consequent inconvenience to authors and readers.

## Supplementary Material

